# Rapid Evolution of Spermathecal Duct Length in the *Allonemobius socius* Complex of Crickets: Species, Population and *Wolbachia* Effects

**DOI:** 10.1371/journal.pone.0000720

**Published:** 2007-08-08

**Authors:** Jeremy L. Marshall

**Affiliations:** Department of Entomology, Kansas State University, Manhattan, Kansas, United States of America; University of Bristol, United Kingdom

## Abstract

The three species in the *Allonemobius socius* complex of crickets have recently diverged and radiated across North America. Interestingly, the only barriers to gene flow between these species in zones of secondary contact appear to be associated with fertilization traits – e.g., conspecific sperm precedence and the ability of males to induce females to lay eggs. Other traits, such as the length of female's reproductive tract, may also influence fertilization success and be associated with species boundaries. However, the underlying variation in this duct has not been assessed across populations and species. Moreover, the effects of reproductive parasites like *Wolbachia* on these morphological features have yet to be addressed, even though its infections are concentrated in reproductive tissues. I evaluated both the natural variation in and the effects of *Wolbachia* infection on spermathecal duct length among several populations of two species in the *Allonemobius socius* complex. My results suggest the following: (1) spermathecal duct length varies between species and is associated with species boundaries, (2) there is considerable variation among populations within species, (3) there is a *Wolbachia* infection-by-population interaction effect on the length of the spermathecal duct, and (4) experimental curing of *Wolbachia* recovers the uninfected morphology. These findings suggest the following hypotheses: (1) spermathecal duct length, like other fertilization traits in *Allonemobius*, is evolving rapidly and influences reproductive isolation and (2) *Wolbachia*-induced modifications of this duct could influence the dynamics of male-female coevolution. Further experiments are needed, however, to explicitly test these latter two hypotheses.

## Introduction

One of the challenges in speciation research is disentangling the traits that diverge after speciation from those that initially drive speciation [1], [2]. For instance, there is a basic relationship between longer times of divergence and greater numbers of traits that isolate species – most of which accumulate long after the speciation event. So, to get at the question of what kinds of traits and processes underlie speciation, it is preferable to study species pairs or complexes that are recently diverged and isolated by only one or a few traits [2]. Several such systems have been identified [e.g. North American field crickets, 3]; [Abalone, 4], [5], including the crickets in the *Allonemobius socius* complex [e.g. 6], [7].

**Figure 1:**
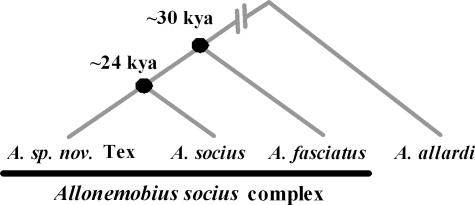
Phylogeny of *Allonemobius socius* complex. Dates of nodes are based on data presented in ref. 7.

The *A. socius* complex of crickets consists of three, cryptic species (*A. socius*, *A. fasciatus*, and *A. sp. nov.* Tex) that likely diverged from a common ancestor within the last 30,000 years (Fig 1; 7). There are also two hybrid zones, one between *A. socius* and *A. fasciatus* that runs latitudinally from New Jersey to central Illinois [8]–[10] and another between *A. socius* and *A. sp. nov.* Tex that runs longitudinally, in the form of a crescent, from southeastern Oklahoma to central Louisiana to southeastern Texas [7]. Much research has shown that the only significant barriers to gene flow between these species pairs are traits related to fertilization [e.g.], [ 1], [ 6], [ 7], [ 11]–[14]. However, it is not a single fertilization trait, but rather a group of fertilization traits that include conspecific sperm precedence [11] and the ability of males to induce females to lay eggs [14] – collectively called gametic isolating traits (see 2, pp 232–246 for a review). In all, the evolution of fertilization traits within populations, whether driven by sexual conflict or sexual selection, underlies mating incompatibilities between heterospecific (or heteropopulation) individuals in this complex.

The importance of gametic isolation in this cricket system suggests that a wide range of fertilization traits may be under selection and thus influence compatibility between different populations or species. One of these traits, the spermathecal duct [a reproductive duct that connects the spermathecae (the sperm storage organ) to the common chamber (a bulbous structure where sperm and accessory gland products are initially deposited during copulation and where sperm are released from during egg-laying & fertilization)], has received a great deal of attention as being a primary player in mediating fertilization success [e.g.], [ 15]–[18]. Additionally, there is overwhelming evidence to suggest a tight coevolution between such female traits and sperm/ejaculate traits [19]–[25]. Miller and Pitnick [24] capture the importance of these structures in the following: “*Likewise, the long and convoluted ducts through which sperm must travel within the female may serve to increase the difficulty for males in placing their sperm close to ova or accessing previously stored sperm, thereby enhancing female control over paternity.”* Taken together, these findings provide strong evidence that characteristics of the female reproductive tract can affect the fertilization success of males.

Given the importance of the length of the female reproductive tract it is critical to understand both its natural variation and the forces that shape its underlying phenotypic distribution – including extrinsic factors such as endosymbionts. One endosymbiont that infects many arthropods is the α-proteobacteria *Wolbachia* [26], [27]. The interest in this dynamic bacterium stems not only from its prevalence and breadth of eukaryotic hosts, but from the wide range of phenotypes that it imposes on its hosts; phenotypes that range from cytoplasmic incompatibility to sex-ratio distortion [27]–[29]. Although *Wolbachia* infections are concentrated in reproductive tissues [e.g.], [30, 31], no one, to my knowledge, has investigated whether or not such infections can modify host reproductive morphology.

Here, I use the *Allonemobius-Wolbachia* host-endosymbiont system [7] to evaluate natural variation in spermathecal duct length. Specifically, I evaluated how this duct varies among populations of two species in the *A. socius* complex (*A. socius* and *A. sp. nov*. Tex) to determine if this particular fertilization trait is evolving rapidly and is associated with species boundaries. Lastly, I assessed the consequences, if any, of *Wolbachia* infection on natural variation in this duct using morphological and molecular techniques and curing experiments.

## Materials and Methods

### Field Sampling

Adults of *A. socius* and *A. sp. nov.* Tex [7] were collected in the field during 2002 and 2003 (Table 1), with all individuals from one location being collected at the same time. Upon returning to laboratory, all individuals (n≈15 per sex) from each population were placed in a population-specific plastic box (20×30×20 cm) with ample saturated cotton. Food (Purina Cat Chow™) was given *ad libitum* and replaced once a week. Cotton was watered to saturation three times a week. These populations were maintained on a 14∶10 light cycle at 27C. A 1∶1 mix of sand and humus soil was provided for females to oviposit and all adults were removed after two weeks. The resulting non-diapause F1's were then raised to adulthood in sex-specific cages (as above) and used for the analyses below.

**Table 1:** Variation in body size and spermathecal duct length among population in the *Allonemobius socius* complex.

Species	Population^a^	Latitude	Longitude	*Wolbachia* Status	N	Body Size mm (SD)	Spermathecal Duct mm (SD)	Allometry
*A. sp. nov.* Tex								
	OK35RA	33.7727	97.1344	UI	6	11.72 (0.71)	8.99 (0.94)	negative
				I	5	11.75 (0.55)	9.79 (0.75)	negative
	TX35494	33.5886	97.1744	UI	13	10.72 (0.56)	8.97 (0.37)	positive
				I	11	11.41 (0.53)	8.66 (0.25)	positive
	TX45241	32.2453	96.4975	UI	11	11.11 (0.91)	8.33 (0.66)	positive
				I	9	11.35 (0.99)	9.39 (0.54)	positive
	TX7575	33.8197	96.5408	UI	4	11.76 (0.81)	9.53 (0.28)	none
				I	3	11.67 (0.38)	8.69 (0.24)	none
*A. socius*								
	AL2038	32.8508	87.9544	UI	6	10.61 (0.43)	8.17 (0.83)	positive
				I	3	10.72 (0.35)	8.22 (0.52)	negative
	NC49HR	35.4847	80.2553	UI	5	12.12 (0.70)	7.68 (0.22)	none
				I	5	11.85 (0.76)	8.54 (0.35)	none

a Population abbreviation as in ref 7. Latitude and longitude are given in decimal degrees. UI = uninfected; I = infected.

### Measurements of body size and spermathecal duct length

Prior to extracting DNA from each female, a digital body size image (ventral side) was taken using a Nikon SMZ 1500 dissecting scope with a DMX 1200 digital camera. The magnification scale of all body size images was the same (7.5X). After taking this image, the posterior portion of the abdomen was removed and stored separately in water at −80°C. The remainder of the abdomen was then used for DNA extraction, while the rest of the body was stored at −80°C.

The spermathecal duct was dissected from the posterior portion of the abdomen. The entire length of the duct, including the spermathecae and common chamber, were dissected to ensure that a standard measurement was recorded for each female. Specifically, the spermathecal duct was placed on a microscope slide in a small drop of water and then held in place by a cover slip. After dissection and mounting, a digital image was taken using the same scope and camera as above. The scale (20X to 50X) of each image was recorded and used to standardize measurements. All images were kept for records. Both body size and spermathecal duct length were measured from images using Image J software from NIH. Measurements were made to the nearest 0.01 mm and done prior to conducting *Wolbachia* screens. For all measurements, the Nikon SMZ 1500 dissecting scope was checked against a know size standard (i.e., 1 mm) approximately every ten measurements. Moreover, all measurements for a given population were taken on the same day and repeated at least twice to ensure an accurate measure. Also, all measures were made independently of *Wolbachia* infection status, so any differences found with regard to infection status can not be attributed to bias in the order of measurement. Once all measurements and *Wolbachia* screens were completed, data sets were combined and analyzed for differences among treatments.

### Statistical analyses of natural variation

First, means and standard deviations of body size and spermathecal duct length were calculated for both infected and uninfected individuals from each population. Moreover, the linear relationship between body size and spermathecal duct length was evaluated for both infected and uninfected individuals in each population. The coefficient of variation (CV) for each trait, across infection status, was also calculated and used in a paired analysis to determine whether or not variation in spermathecal duct length was greater than that of body size. CV was calculated using the sample-size corrected formula (see 32 for details): (1+1/4*n*) (*s*/*u̅*)(100), where *n* is sample size, *s* is the standard deviation and *u̅* is the mean. Specifically, once a CV pair was generated for each population a binomial scoring system was applied to each pair (1 = CV of spermathecal duct length is greater than CV of body size, 0 = CV of spermathecal duct length is less than, or equal to, CV of body size). A binomial distribution was then used to determine the exact probability of getting *j*-number of 1's out of *n*-number of population pairs.

Next, mixed model nested ANOVAs were conducted on both body size and spermathecal duct length with infection status as a fixed factor and population (random factor) being nested within species (fixed factor). Because spermathecal duct length can vary with body size, I combined all data and regressed spermathecal duct length against body size to generate body-size free residuals of spermathecal duct length. These residuals were used in the nested ANOVA. Both body size and spermathecal duct length met the assumptions of ANOVA. Alpha was set at 0.05 for all comparisons. All ANOVAs were carried out using PROC GLM in SAS 9.1 (2002–2003 by SAS Institute Inc., Cary, NC, USA).

### Screening for Wolbachia

The *Wolbachia* strain infecting these populations, and most other populations in the southern United States [7], is a haplotype of *w*Con that is very similar to *w*Con found in the beetle, *T. confusum*. For example, these two haplotypes have 96.1 amino acid similarity, respectively, based on *wsp* gene sequences (GenBank accession nos.: *A. socius* host, AY705232; *T. confusum* host, AF020083).

As for screening, genomic DNA isolated from each individual was used as the template in a PCR test for *Wolbachia*. All maternal females and F1 offspring were screened for *Wolbachia* using the universal primers ftsZUNIF and ftsZUNIR that amplify an ≈750 bp fragment of the bacterial cell-cycle gene, *ftsZ* [33]. The thermocycler profile followed Bandi et al. [34]. Following PCR, samples were run on a 1% agarose gel and visualized. The *Drosophila simulans* Riverside strain of *Wolbachia* was used as a positive control (complements of William Ballard via Bryant McAllister; both of whom are currently at The University of Iowa). A control for host DNA, such as a nuclear or mitochondrial gene, was not conducted for any sample.

### Curing Experiment

Adults of *A. sp. nov.* Tex were collected in the field from TX35494 and brought back to my laboratory during September of 2003. All individuals (n≈15 per sex) from this population were maintained as above. Within the population cage, females were allowed to mate for 7 days. After this seven day period all females were removed and placed in individual egg-laying chambers. Each egg-laying chamber had a 4 cm long, loose role of saturated cheese cloth (1 cm diameter) for use as an egg-laying substrate. Each female was allowed to lay eggs for two weeks at 27°C. Females were then stored at -80°C and later screened for *Wolbachia* using the *ftsZ* gene.

Hatchlings from each female were raised to adulthood in separate plastic containers under the same conditions as above. However, half of the F1 hatchlings for each female were placed in a separate plastic container and given cotton saturated with tetracycline treated water (2 g/L). The above treatment was done regardless of infection status. This experimental manipulation resulted in two classes of treatment: i) infected females that gave rise to both infected and cured offspring and ii) uninfected females that gave rise to uninfected offspring that either received or did not receive antibiotics. These treatments allowed me to test the effects of *Wolbachia* infection and antibiotics on reproductive morphology, while controlling for genetic background.

### Statistical analyses for curing experiment

Before testing for differences in spermathecal duct length among infected, cured, and naturally uninfected females, I statistically assessed if data from different female lines, yet from the same treatment, could be combined for further analysis. To accomplish this, I used ANCOVA in conjunction with power analysis for various effect sizes. Effect sizes of 0.3 and 0.5 mm in duct length were chosen, as these reflect spermathecal duct length differences of approximately 3 and 5%, respectively (given average duct length is ∼9.0 mm). Additionally, the actual effect size per comparison was calculated by using least square mean (LSM) duct lengths. Power was evaluated with the online freeware ‘Power Analysis for ANOVA Designs’ by M. Friendly (www.math.yorku.ca/ SCS/online/power) using mean square error (MSE) and average sample size per comparison (rounding down where appropriate to the nearest whole integer).

Following the above analyses, data from different lines within each treatment were combined and used to assess treatment group effects. Specifically, treatment groups (i.e., infected, cured, and naturally uninfected females) were analyzed with an ANCOVA, while controlling for the influence of body size (i.e., treatment group as the independent variable, spermathecal duct length as the dependent variable, and body size as the covariate). Post-hoc ANCOVAs were then used to determine which treatment groups differed from one another. Alpha was set at 0.05 for all comparisons. All ANCOVAs were carried out using PROC GLM in SAS 9.1.

## Results

### Results of natural variation

Means, standard deviations, and the linear relationship between body size and spermathecal duct length are presented in Table 1. There is incredible diversity in the allometry between body size and spermathecal duct length, ranging from positive to negative slopes (Table 1). As for whether or not there is greater variation in spermathecal duct length than body size, I found that CV_spermathecal duct length_ is on average 36% greater than CV_body size_, although these measures are not significantly different from one another (*P*_paired-binomial, n = 6_ = 0.109; CV_body size_ was greater than CV_spermathecal duct length_ in only TX35/494).

**Figure 2:**
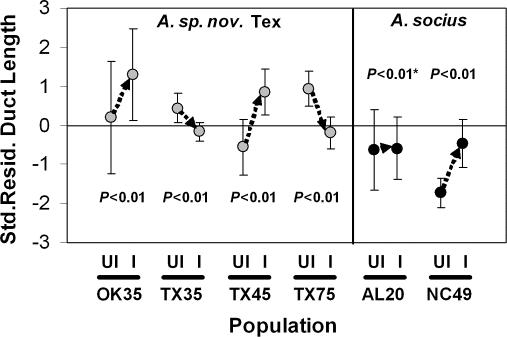
Species and *Wolbachia* effects on spermathecal duct length. UI = uninfected, I = infected. Population abbreviations are based on population names presented in Table 1. * = significant interaction effect in the within population ANCOVA. Standard deviation bars are presented.

The nested ANOVA for body size revealed a significant population(species) effect, suggesting that body size varies among populations within a species (*P* = 0.0002; Table 2). However, no other effects were significant, including the effect of *Wolbachia* infection (*P* = 0.4971; Table 2). As for residual spermathecal duct length, there was a significant species effect, with *A. sp. nov*. Tex having longer ducts, on average, than *A. socius* (*P* = 0.0138; Table 3, Fig. 2). There was also a significant population X infection interaction effect, indicating that *Wolbachia* infections can either result in a shortening or lengthening of the spermathecal duct depending on host genetic background or differences among strains (*P*<0.0001; Table 3, Fig. 2). Post-hoc ANCOVAs within each population showed that *Wolbachia* infections modified the length or allometry of the spermathecal duct in all populations (Fig. 2; independent variable was infection status, dependent variable was spermathecal duct length, and the covariate was body size). The median change, whether longer or shorter, in spermathecal duct length as a consequence of *Wolbachia* infection was 9.3% longer or shorter, respectively, than the uninfected length; this is equivalent to a 1 SD shift in spermathecal duct length.

**Table 2:** Mixed model nested ANOVA on body size.

Source	DF	SS	MS	*F*	*P*
Species	1	0.16	0.16	0.05	0.8319
Infect	1	0.23	0.23	0.47	0.4971
Pop(Species)	4	12.36	3.09	6.26	0.0002*
Pop×Infect(Species)	5	2.18	0.44	0.88	0.4964
Error	69	34.07	0.49		

Population (random factor) is nested within species (fixed factor), as is the population×infection interaction effect. Infect = infection status (fixed factor);

* = signifcant at alpha = 0.05.

**Table 3:** Mixed model nested ANOVA on residual spermathecal duct length.

Source	DF	SS	MS	*F*	*P*
Species	1	18.852	18.95	17.57	0.0138*
Infect	1	1.911	1.91	3.69	0.0588
Pop(Species)	4	4.291	1.07	2.07	0.0937
Pop×Infect(Species)	5	18.467	3.69	7.14	<0.0001*
Error	69	35.705	0.52		

Population (random factor) is nested within species (fixed factor), as is the population×infection interaction effect. Infect = infection status (fixed factor);

* = signifcant at alpha = 0.05.

### Results of curing experiment

With regard to the effects of antibiotics on naturally uninfected lines, data from two isofemale lines, whereby half the hatchings from a particular female were given antibiotics and the other half were not, indicate that antibiotics do not influence the length of the spermathecal duct (*P = *0.615; difference in LSM duct length = 0.06 mm; Table 4). This result suggests that the antibiotic is not responsible for the results presented here. Therefore, data from all naturally uninfected F1 offspring were combined for the analysis of treatment groups.

**Table 4:** Descriptive statistics and comparisons of female lines within treatment groups.

Comparison	Descriptive Statistics				Analysis			Minimum Power a	
								Difference in Duct Length	
	N	Body Length (SD)	Duct Length (SD)	LSM Duct Length	*MSE*	*F*	*P*	0.3 mm	0.5 mm
1. Naturally Uninfected									
(A) antibiotics vs.	7	10.745 (0.722)	9.010 (0.406)	8.998	0.0425	0.270	0.615	0.623	0.985
(B) no antibiotics	6	10.707 (0.444)	8.929 (0.356)	8.938					
2. Infected									
(A) line U vs.	8	11.438 (0.568)	8.679 (0.290)	8.669	0.0200	0.040	0.845	0.837	0.998
(B) line AA	3	11.342 (0.533)	8.621 (0.132)	8.650					
3. Cured									
(A) line V vs.	6	10.786 (0.523)	8.825 (0.273)	8.800	0.0173	6.940	0.030*	0.882	0.999
(B) line BB	5	10.659 (0.936)	8.979 (0.431)	9.010					

a Power analyses based on MSE and sample size.

* = significant at the alpha = 0.05 level.

As for infected and cured lines, two infected females, that produced both infected and cured (uninfected) lines, were used in this study. These individuals gave rise to two cured lines (named V and BB) and two infected lines (named U and AA). Lines AA and BB were derived from one infected female, while lines U and V were derived from the other. Data from the two infected lines did not significantly differ from one another (*P = *0.845; difference in LSM duct length = 0.019; Table 4). However, the 0.21 mm difference in LSM duct length between cured lines was significant (*P = *0.03; Table 4). This latter result indicates that group sample sizes of 5 can detect alterations in spermathecal duct length as small as ±2%. Overall, power analyses suggest that my between line, within treatment sampling is sufficient to detect modifications of ±2–5% (Table 4). As for combining data, I did so for all lines within a given treatment for use in the analysis of treatment groups. Although the individuals from cured line V have slightly shorter ducts than line BB or naturally uninfected individuals, combining data from lines V and BB (the cured lines) made my analysis of treatment groups more conservative, as *Wolbachia* infections appear to shorten the length of the spermathecal duct in the TX 35/494 population (Table 1, Fig. 2). Also, as stated in the methods, the infection status of all individuals used in this analysis was confirmed with PCR tests.

**Figure 3:**
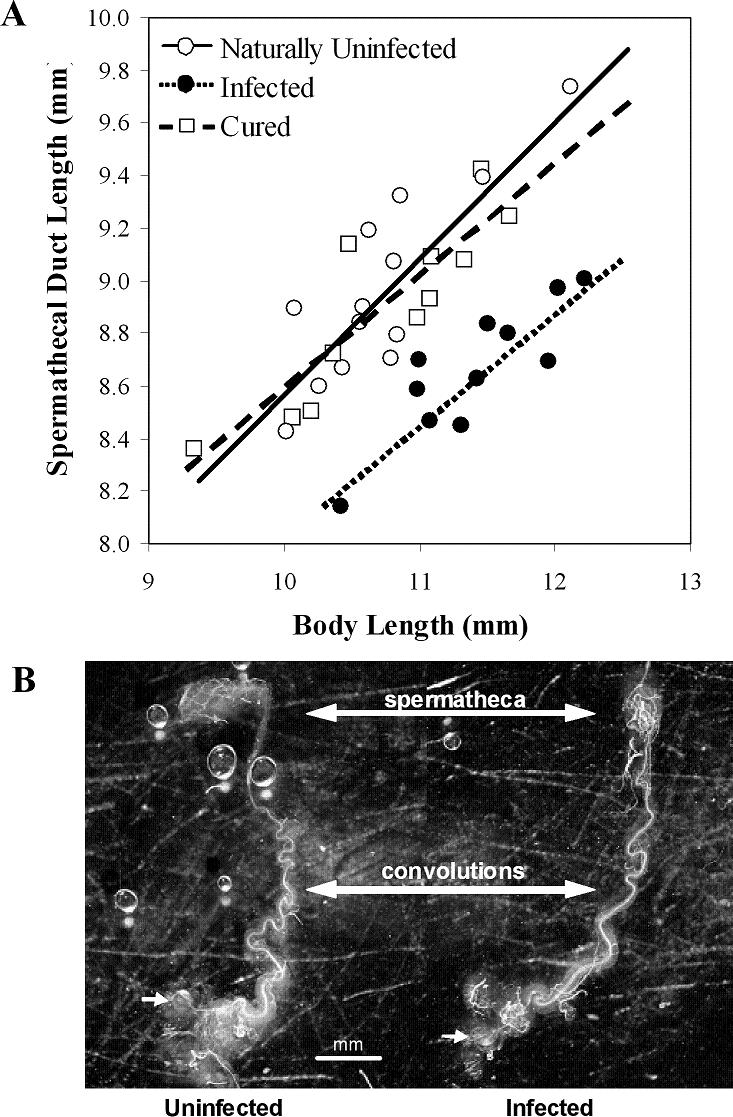
Wolbachia effects on spermathecal duct length. (A) The relationship between body size and spermathecal duct length for *Wolbachia* infected (black), cured (open square), and naturally uninfected (open circle) females. Infected females are significantly different from cured and naturally uninfected females (see text). (B) Representative spermathecal ducts from an uninfected and infected female with nearly identical body sizes (10.63 and 10.42 mm, respectively). The small arrows show the common chamber where sperm enter the spermathecal duct during copulation and where the sperm leave upon fertilization. The large double-headed arrows identify convolutions (or lack thereof) and the balloon-shaped spermatheca (where sperm are stored until fertilization).

The analysis of treatment groups revealed that *Wolbachia* infections significantly reduced the length of the spermathecal duct across all body sizes (*P*<0.0001; Table 5; Fig. 3A). The loss of length is apparent in Fig. 3B, where the high degree of convolution is lacking in the female reproductive tract infected by *Wolbachia*. Moreover, post-hoc ANCOVAs revealed no differences in duct length between cured and naturally uninfected females (*F*_1, 21 = _0.91, *P = *0.3513), while both of the latter treatment groups yielded significant treatment effects when compared to data from infected females (*F*_1,19 = _62.67, *P*<0.0001 and *F*_1, 21 = _53.02, *P<*0.0001, respectively). There were also no slope differences between any treatments (i.e., no interaction effects). In total, the spermathecal duct of infected females was 0.55 and 0.62 mm shorter than that of cured or naturally uninfected females, respectively. After controlling for body size, this is equivalent to an average reduction of 0.6 SD in the length of the female reproductive tract.

**Table 5:** *Wolbachia* curing experiment ANCOVA.

Source	DF	SS	MS	*F*	*P*	Power
Treatment	2	2.0351	1.0175	34.95	<0.0001	0.999
Body Size	1	2.5107	2.5107	86.23	<0.0001	
Error	31	0.9026	0.0291			

## Discussion

This study was conducted to determine if female reproductive morphology, like other fertilization traits (e.g., conspecific sperm precedence), is associated with species boundaries in the rapidly evolving *Allonemobius socius* complex of crickets. Results presented here show that the length of the female reproductive tract (i.e., spermathecal duct length) is associated with species boundaries, with female *A. sp. nov.* Tex having longer tracts than females of *A. socius*. Although these two species are separated by only about twenty-four thousand years and exhibit few behavioral or ecological differences, there is strong genetic bimodality in their zone of overlap, 78 to 90% conspecific sperm precedence [7], and heterospecific males are significantly less successful, relative to conspecifics, at inducing females to lay eggs [7], [11; Marshall unpub. data]. Together, along with decades of research on the *A. socius-A. fasciatus* hybrid zone [1], [6], [8]–[14], these data suggest that fertilization traits as a whole are evolving rapidly in this cricket group.

The finding that selection appears to be shaping a diverse array of traits, including seminal fluid proteins [35], sperm-reproductive tract interactions [11], and the length of the female's reproductive tract (this study), related to one kind of reproductive isolating barrier is not novel, but it does say something about the relative importance of barriers to fertilization in the evolution of new species in this complex of crickets – specifically, barriers to fertilization are the primary traits underlying speciation in the *A. socius* complex. In a time when evolutionary biologists are concerned with questions such as “which kinds of traits are most important in speciation”, “do certain kinds of traits tend to evolve before others” and “what is the relative importance of processes like sexual conflict and ecological selection” (see ref. 2), the *A. socius* complex would appear to be adding a data point to this conversation.

As for factors that underlie natural variation in spermathecal duct length, there are population-specific and *Wolbachia* effects in addition to the species-level differences. Interestingly, there is a population×*Wolbachia* infection status interaction nested within the species-level effect. This is not only the first time that *Wolbachia* has been suggested to modify the length of the female reproductive tract, but the type of modification appears to depend on the genetic background of the population and the exact strain of *Wolbachia* (Table 1, 2, and 3; Fig. 2). For example, the curing experiment shows that *Wolbachia* infections shorten the length of spermathecal duct in the TX35/494 population of *A. sp. nov.* Tex (Fig. 3), a pattern seen in at least one other natural population (Fig. 2). However, *Wolbachia* infections appear to increase the length of the spermathecal duct in several other populations of both species. Given that the length of female reproductive tract has been implicated in male fertilization success and sperm competition [e.g.], [25], [36, 37], this proposed *Wolbachia*-induced modification has the potential to influence the dynamics of fertilization success and even male-female coevolution.

A caveat to the above results presented for *Wolbachia* is that a control PCR for host DNA was not conducted, so these results could somehow be biased by the ability to amplify host and *Wolbachia* DNA. However, given the consistent pattern that “infected” individuals always differed from “uninfected” individuals within a population, the results presented here do suggest an association between some strain of infectious agent and host spermathecal duct length. Moreover, it is still possible that another bacterium, other than *Wolbachia*, underlies these differences – as another bacterium could co-occur with *Wolbachia*. Although unlikely, this hypothesis has not been eliminated and awaits further testing.

Regardless of the underlying bacterium, the existence of this phenotype raises an intriguing question – how could this phenotype help maintain an endosymbiotic infection within a population? The usual *Wolbachia*-induced phenotypes of cytoplasmic incompatibility and sex-ratio distortion [27]–[29] are easy to explain, as the bacteria's selfish habits convey a fitness advantage to females; thus resulting in the maintenance of infection. In this system, these latter two phenotypes do not exist (7; pers. obs.), so we are left with the above question. One possibility is that *Wolbachia*-induced modifications of the female reproductive tract interplay with the dynamics of sexual conflict resulting in the maintenance of *Wolbachia* infections – i.e., within the dynamics of sexual conflict, *Wolbachia*-infected females have higher fitness than their uninfected counterparts. Such interplay could also result in the evolution of reproductive isolation. Specifically, the interaction between host genetic background and strain-type could alter the trajectory of male-female antagonistic coevolution within populations resulting in postmating, prezygotic incompatibilities upon secondary contact. Although conceptually possible, this hypothesis needs to be analytically modeled and empirically tested in a more rigorous framework.
